# Association Between ALDH-2 rs671 and Essential Hypertension Risk or Blood Pressure Levels: A Systematic Review and Meta-Analysis

**DOI:** 10.3389/fgene.2020.00685

**Published:** 2020-07-15

**Authors:** Yawei Zheng, Cheng Ning, Xingxing Zhang, Yuhao Zhao, Yizhuo Li, Lichao Qian, Jie Li, Zhuyuan Fang

**Affiliations:** ^1^Affiliated Hospital of Nanjing University of Chinese Medicine, Nanjing, China; ^2^Nanjing University of Chinese Medicine, Nanjing, China

**Keywords:** ALDH-2, rs671, essential hypertension, blood pressure, meta-analysis, polymorphism

## Abstract

**Background:** The association between Aldehyde dehydrogenase II (ALDH-2) rs671 polymorphism and essential hypertension (EH) risk or blood pressure (BP) levels remains unclear.

**Objective:** To systematically review the influence of the aldehyde dehydrogenase II rs671 polymorphism on essential hypertension risk and blood pressure levels.

**Methods:** The PubMed, EMbase, Web of Science, Cochrane Library, CNKI and CBM databases were electronically searched to identify case-control or cohort studies published prior to July 2019 that examined the association between the rs671 polymorphism and the risk of essential hypertension or blood pressure levels. A meta-analysis was conducted with Stata 15.1 software.

**Results:** Twenty-two articles were included. Among these articles, 20 incorporated 30 individual studies evaluating the association between the rs671 polymorphism and EH (11,051 hypertensive patients and 15,926 normotensive controls), and 8 incorporated 12 individual studies evaluating the association between the rs671 polymorphism and BP (20,512 subjects). The results of the meta-analysis showed that the mutation of the rs671 polymorphism was associated with a significantly decreased risk of EH in all models: allelic model (OR = 0.80, 95% CI: 0.73–0.87), homozygous model (OR = 0.71, 95% CI: 0.63–0.80), heterozygous model (OR = 0.79, 95% CI: 0.72–0.87), dominant model (OR = 0.79, 95% CI: 0.71–0.87), and recessive model (OR = 0.76, 95% CI: 0.68–0.85). In the stratified analyses, significant associations were found for males, drinkers and population-based studies. Simultaneously, the A carriers had lower SBP (WMD = −1.78, 95% CI: −3.02 to −0.53) and DBP (WMD = −1.09, 95% CI: −1.58 to −0.61) levels than individuals with the GG homozygote.

**Conclusion:** The collective findings of this meta-analysis suggested that the ALDH-2 rs671 polymorphism represented an important genetic marker in the development of hypertension. Considering the overall quality of evidence and the relatively small pooled sample size, more well-conducted high-quality studies are required to verify the above conclusion.

**Systematic Review Registration Number:** PROSPERO (CRD42019129746).

## Highlights

- The rs671 polymorphism was associated with essential hypertension risk.
- The rs671 polymorphism was associated with blood pressure levels.
- The rs671 polymorphism represents an important genetic marker of hypertension.

## Introduction

Hypertension is one of the most common chronic non-infectious diseases and is recognized as a major causal risk factor for cardiovascular diseases. Elevated blood pressure is the leading cause of death worldwide, and the burden of hypertension is expected to increase globally. In 2012, hypertension affected 270 million individuals in China and had a prevalence of 25.2% (Chen et al., 2018), which significantly increased with age. In 2013, hypertension alone accounted for 6.61% of the 3.1869 trillion RMB spent on healthcare in China (Chen et al., 2017). In Japan, the mean blood pressure has steadily declined over the past 50 years, but hypertension remains one of the biggest risk factors for non-communicable diseases, particularly cardiovascular disease (Ikeda et al., 2011; Lim et al., 2012).

Hypertension is a disease whose pathophysiological mechanism involves hundreds of genes (Hwang et al., 2012). Numerous epidemiological studies have elucidated some risk factors, such as sex, age, and drinking alcohol (Kario, 2015). Previous studies have investigated the association between gene polymorphisms and hypertension (Ma et al., 2016; Niu et al., 2019). The aldehyde dehydrogenase (ALDH) super family includes key enzymes in the major pathway of alcohol metabolism. Aldehyde dehydrogenase 2 (ALDH-2) has a critical role in mediating the conversion of aldehydes into much less reactive chemical species (Xu et al., 2017). Several studies have shown that ALDH-2 deletion is a susceptibility factor for blood pressure levels and could increase oxidative stress (Ohsawa et al., 2003). The mutation in exon 12 in which G is changed to A (rs671, Glu504Lys) resulted in decreased enzyme activity (Perez-Miller et al., 2010), thereby affecting blood acetaldehyde concentrations after alcohol intake (Eriksson, 2001). Recently, numerous published studies have confirmed that the ALDH-2 rs671 polymorphism is associated with hypertension (Ota et al., 2016; Du, 2018). However, the evidence remains inconclusive. Other studies have found that ALDH-2 rs671 polymorphism is not associated with hypertension (Wu et al., 2013; Zhang et al., 2016). Whether there is a relationship between the ALDH-2 rs671 polymorphism and hypertension remains unclear. Even previous meta-analysis studies on this issue have drawn different conclusions, and only focused on the association between ALDH-2 and essential hypertension risk (Jia et al., 2015; Li et al., 2017; Wu et al., 2017). In order to conclude the influence of the ALDH-2 rs671 polymorphism on hypertension, we performed a more comprehensive systematic review and meta-analysis. Furthermore, we not only estimate the association between the ALDH-2 rs671 polymorphism and essential hypertension risk, but also estimate the association between the ALDH-2 rs671 polymorphism and blood pressure levels. At the same time, we carried out a series of subgroup analysis to make the result more practical. This study was registered with PROSPERO (CRD42019129746) and performed according to the PRISMA (Preferred Reporting Items for Systematic Reviews and Meta-analyses) guidelines (Liberati et al., 2009).

## Materials and Methods

### Literature Search

To be as comprehensive as possible, two authors independently performed a systematic search of six available electronic databases, including PubMed, EMbase, Web of Science (WOS), the Cochrane Library, Chinese Biomedical Literature Database (CBM) and China National Knowledge Infrastructure (CNKI), for studies published prior to July 2019. The keywords included “hypertension,” “essential hypertension,” “EH,” “blood pressure,” “aldehyde dehydrogenase 2,” “ALDH 2,” “ALDH-2,” “rs671,” “genotype,” “alleles,” “polymorphism,” “mutation,” and “variation.” We used both MeSH terms and Title/Abstract search. Languages were not restricted during the searching process. Published articles listed in the references of the keyword index results were also screened carefully to avoid possible omissions.

### Inclusion and Exclusion Criteria

Studies were included if they fulfilled all of the following criteria: (i) case-control design or cohort study design; (ii) studies that examined the association between the ALDH-2 rs671 polymorphism and the risk of essential hypertension or blood pressure levels; (iii) diagnosis of hypertension defined as either systolic blood pressure ≥140 mmHg or diastolic blood pressure ≥90 mmHg (continuously or more than 3 times in a sitting position, on three different days), or taking antihypertensive medication. The exclusive criteria were as follows: (i) repeated publication of literature or reported duplicate data; (ii) research that is not available in full text; and (iii) reports with incomplete data or no usable data.

### Data Extraction

A data-extraction table was designed in Excel 2016 by all the researchers. The following information was included: name of the first author, year of publication, ethnicity of study population, study design, source of population, genotyping methodology, sample size, average age, gender, alcohol consumption, SBP and DBP. Two researchers independently extracted the necessary data, and all disagreements were resolved through discussion with a third researcher.

### Quality Assessment

Two researchers evaluated the quality of each included study using the Newcastle-Ottawa Scale (NOS) (Stang, 2010) independently. Three major aspects of study quality were scored: (i) selection of the study groups (0–4 scores); (ii) determination of the exposure of interest in the studies (0–3 scores); and (iii) the quality of the adjustment for confounding variables (0–2 scores). Scores ranged from zero to nine stars, and a score of six or above was considered a high-quality study.

### Statistical Analysis

The χ^2^ test was used to assess whether the genotype distributions in the control group of each study were in Hardy-Weinberg Equilibrium (HWE). Heterogeneity among the same category was evaluated using the χ^2^ test and Cochran's Q statistic, and the *I*^2^ statistic was used to quantify the percentage variability of the heterogeneity (Higgins et al., 2003). If *P* > 0.1 and *I*^2^ ≤ 50%, the fixed-effect model was selected; otherwise, the random-effect model was adopted (DerSimonian and Laird, 1986). The risk of EH was estimated by the pooled odds ratio (OR) along with the 95% confidence interval (CI), and the levels of BP was signified as the weighted mean difference (WMD) with its 95% CI. The pooled ORs and WMDs were measured using the *Z*-test, and a *P*-value of < 0.05 was considered statistically significant. A sensitivity analysis was performed to detect the individual effect of each study on the pooled ORs or WMDs by omitting one individual inter-study at a time. Publication bias was evaluated with Begg's test, Egger's test and the trim-and-fill method. All statistical analyses were performed with Stata 15.1 software.

## Results

### Characteristics of Included Studies

**Figure 1:**
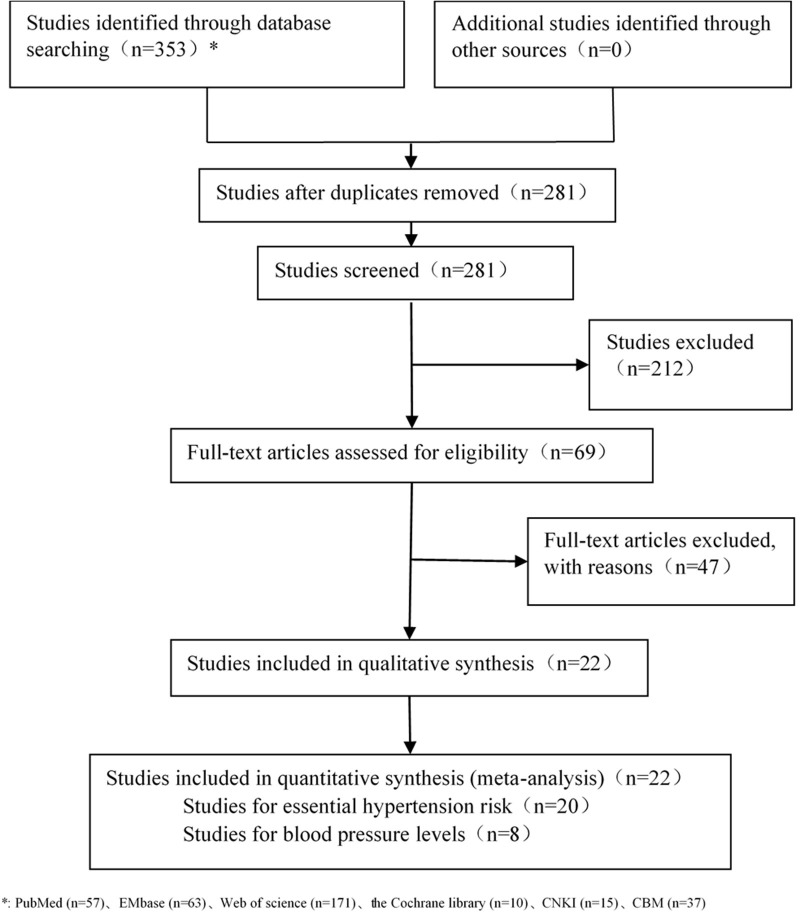
Flow chart for literature screening.

Two researchers independently sifted through the literature, extracted the data and cross-checked them. In case of disagreement, the decision was made through discussion or arbitration by the third researcher. A total of 353 articles were found after searching the existing literature electronic databases. After removing duplicates, 281 articles were retained. A total of 212 articles with irrelevant data were excluded after a further review of titles and abstracts. The full texts of the remaining articles were screened carefully, and another 47 articles were excluded. Finally, 22 articles were included. The selection process for the qualified publications is presented in Figure 1.

Finally, 22 articles (Takagi et al., 2001; Amamoto et al., 2002; Saito et al., 2003; Iwai et al., 2004; Hui et al., 2007; Hasi et al., 2011; Feng et al., 2012; Lv et al., 2013; Nakagawa et al., 2013; Wang et al., 2013; Wu et al., 2013, 2017; Yokoyama et al., 2013; Zhang et al., 2013, 2016, 2018; Isomura et al., 2015; Jing et al., 2015; Ota et al., 2016; Li et al., 2017; Ma et al., 2017; Du, 2018) met the preset inclusion criteria. Among these articles, 20 (Takagi et al., 2001; Amamoto et al., 2002; Saito et al., 2003; Iwai et al., 2004; Hui et al., 2007; Hasi et al., 2011; Feng et al., 2012; Lv et al., 2013; Nakagawa et al., 2013; Wang et al., 2013; Wu et al., 2013, 2017; Yokoyama et al., 2013; Jing et al., 2015; Ota et al., 2016; Zhang et al., 2016, 2018; Li et al., 2017; Ma et al., 2017; Du, 2018) assessed the association between the rs671 polymorphism and EH risk in 30 individual studies (11,051 hypertensive patients and 15,926 normotensive controls), and 8 (Takagi et al., 2001; Amamoto et al., 2002; Saito et al., 2003; Wang et al., 2013; Zhang et al., 2013; Isomura et al., 2015; Ota et al., 2016; Li et al., 2017) assessed the association between the rs671 polymorphism and BP levels in 12 individual studies (20,512 subjects). The baseline characteristics of all included articles are summarized in Table 1 (for EH) and Table 2 (for BP). For the EH association articles, the genotype distribution in the control groups was not in line with HWE in two articles (Yokoyama et al., 2013; Du, 2018), and in another two articles (Nakagawa et al., 2013; Ota et al., 2016) HWE could not be tested. The NOS scores of all studies were six or higher, indicating that they were high-quality studies. For the BP association articles, we extracted the blood pressure data of rs671A variant carriers. The NOS scores of all studies were higher than six, indicating that they were high-quality studies.

**Table 1:** The baseline characteristics of all included articles (for EH).

**Study**	**Ethnicity**	**Genotyping method**	**Source**	**Study design**	**Size**	**Alcohol consumption**	**Gender**	**Case**			**Control**			**HWE**	**NOS**
								**GG**	**AG**	**AA**	**GG**	**AG**	**AA**		
Amamoto et al. (2002)	Japan	PCR-RFLP	P	Cohort study	2035	Mixed	Overall	395	342	51	584	564	99	0.2020	7
						Mixed	Male	161	134	17	174	217	46		
						Mixed	Female	234	208	34	410	347	53		
(Du, 2018)	China	PCR-RFLP	H	Case-control	337	Mixed	Overall	112	30	2	114	77	2	0.0048	8
Feng et al. (2012)	China	PCR-RFLP	H	Case-control	111	Mixed	Overall	53	26	1	17	12	2	0.9517	7
						Mixed	Male	45	22	0	13	9	2		
						Mixed	Female	8	4	1	4	3	0		
Hasi et al. (2011)	China	TaqMan PCR	P	Case-control	161	Mixed	Overall	83	8	0	55	15	0	0.3154	6
						Mixed	Male	38	6	0	32	5	0		
						Mixed	Female	45	2	0	23	10	0		
Hui et al. (2007)	Japan	TaqMan PCR	P	Case-control	532	Mixed	Overall	166	81	14	136	114	21	0.6674	6
						Mixed	Male	118	45	7	90	78	14		
						Mixed	Female	36	48	7	46	36	7		
Iwai et al. (2004)	Japan	TaqMan PCR	P	Cohort study	1852	Mixed	Overall	413	300	51	550	429	109	0.0630	8
						Mixed	Male	220	151	22	223	197	45		
						Mixed	Female	193	149	29	327	232	64		
Jing et al. (2015)	China	TaqMan PCR	P	Case-control	832	Mixed	Overall	338	126	10	220	122	16	0.8605	7
						Mixed	Male	200	80	6	136	88	9		
						Mixed	Female	138	46	4	84	34	7		
Li et al. (2017)	China	TaqMan PCR	P	Case-control	3038	Mixed	Overall	1138	691	94	653	390	72	0.1848	8
Lv et al. (2013)	China	PCR-RFLP	H	Case-control	465	Mixed	Overall	73	30	2	209	139	12	0.0522	8
Ma et al. (2017)	China	PCR	H	Case-control	4018	Mixed	Overall	871	295	15	1888	857	92	0.6613	6
Nakagawa et al. (2013)	Japan	PCR-RFLP	H	Cohort study	444	Mixed	Overall	74	49		171	150		N	7
						Drinkers	Overall	54	25		121	53			
						Nodrinkers	Overall	20	24		50	97			
Ota et al. (2016)	Japan	PCR-RFLP	P	Case-control	1225	Mixed	Male	137	62		630	396		N	7
Saito et al. (2003)	Japan	PCR-RFLP	P	Cohort study	335	Drinkers	Male	77	44	3	100	93	18	0.5776	8
Takagi et al. (2001)	Japan	TaqMan PCR	P	Cohort study	4057	Drinkers	Overall	809	598	133	1227	1065	225	0.7782	8
						Drinkers	Male	421	289	63	503	536	107		
						Drinkers	Female	388	309	70	724	529	118		
Wang et al. (2013)	China	PCR-LDR	P	Case-control	2119	Mixed	Overall	668	373	57	560	396	65	0.6531	7
						Drinkers	Overall	166	40	3	135	67	3		
						Nondrinkers	Overall	502	333	54	425	329	62		
Wu et al. (2013)	China	PCR-RFLP	H	case-control	737	Mixed	Overall	254	59	8	353	58	5	0.1468	6
Wu et al. (2017)	China	PCR-LDR	P	Case-control	2326	Mixed	Overall	586	440	65	606	531	98	0.2181	8
						Mixed	Male	267	206	33	264	250	50		
						Mixed	Female	319	234	32	342	281	48		
Yokoyama et al. (2013)	Japan	PCR-RFLP	P	Cohort study	1902	Drinkers	Male	433	62	0	1172	235	0	0.0006	7
Zhang et al. (2016)	China	PCR	P	Case-control	212	Mixed	Overall	95	17	0	86	13	1	0.5283	7
Zhang et al. (2018)	China	PCR-RFLP	H	Case-Control	239	Nodrinkers	Overall	80	39	18	71	26	5	0.2141	7

**Table 2:** The baseline characteristics of all included articles (for BP).

**Study**	**Ethnicity**	**Alcohol consumption**	**Gender**	**size**				**SBP**				**DBP**				**NOS**
				**GG**	**AG**	**AA**	**A carriers**	**GG**	**AG**	**AA**	**A carriers**	**GG**	**AG**	**AA**	**A carriers**	
Amamoto et al. (2002)	Japan	Mixed	Male	335	351	63	414	134.2 ± 18.5	131.8 ± 18.2	125.8 ± 17.4	130.89 ± 18.56	79.9 ± 11.7	78.2 ± 11.2	75.5 ± 11.2	77.79 ± 11.23	7
			Female	644	555	87	642	127.2 ± 20.4	127.4 ± 19.9	128.1 ± 18.7	127.49 ± 19.73	75.1 ± 11.7	75.5 ± 11.4	76.7 ± 11.7	75.66 ± 11.44	
Isomura et al. (2015)	Japan	Mixed	Overall	1947	1255		1255	134.0 ± 0.6	132.2 ± 0.6		132.2 ± 0.6	81.6 ± 0.3	80.3 ± 0.4		80.3 ± 0.4	7
Li et al. (2017)	China	Mixed	Overall	1791	1081	166	1247	143.2 ± 22.7	142.1 ± 22.4		142.1 ± 22.4	81.1 ± 11.4	81.0 ± 11.4		81.0 ± 11.4	8
Ota et al. (2016)	Japan	Mixed	Male	767	458		458	125.5 ± 13.7	122.2 ± 13.6		122.2 ± 13.6	78.6 ± 10.5	76.0 ± 11.0		76.0 ± 11.0	7
Saito et al. (2003)	Japan	Mixed	Male	177	137	21	158	132.1 ± 21.5	127.6 ± 20.1	118.2 ± 13.4	126.35 ± 19.57	81.6 ± 10.2	78.4 ± 11.0	74.5 ± 10.5	77.88 ± 10.98	8
Takagi et al. (2001)	Japan	Mixed	Male	924	825	170	995	132.0 ± 0.7	128.3 ± 0.7	126.1 ± 1.5	127.92 ± 1.21	82.7 ± 0.4	80.6 ± 0.4	79.6 ± 0.8	80.43 ± 0.62	8
			Female	1112	838	188	1026	128.2 ± 0.6	129.5 ± 0.7	128.3 ± 1.5	129.28 ± 1.01	79.6 ± 0.8	78.8 ± 0.4	79.0 ± 0.3	78.84 ± 0.39	
Wang et al. (2013)	China	Drinkers	Overall	301	113		113	132.0 ± 18.9	126.5 ± 15.0		126.5 ± 15.0	82.6 ± 10.9	81.3 ± 9.3		81.3 ± 9.3	7
		Non-drinkers		927	778		778	129.8 ± 19.0	128.6 ± 19.0	128.6 ± 19.0		80.6 ± 10.5	79.9 ± 10.5		79.9 ± 10.5	
Zhang et al. (2013)	China	Drinkers	Overall	1242	717	85	802	133.5 ± 1.8	133.2 ± 2.1	130.7 ± 4.6	132.94 ± 2.60	77.5 ± 1	76.7 ± 1.1	76.1 ± 2.4	76.64 ± 1.31	8
		Non-drinkers		969	1173	315	1488	132.7 ± 1.4	132.9 ± 1.4	132.2 ± 2.3	132.75 ± 1.66	75.1 ± 0.8	75.1 ± 0.7	74.5 ± 1.3	74.97 ± 0.90	

### Meta-Analysis for the Risk of Essential Hypertension: Integral Analyses

The risk prediction of the rs671 polymorphism for essential hypertension was investigated separately under the allelic, homozygous, heterozygous, dominant and recessive models. The detailed results of the ORs and 95% CIs for different comparisons are shown in Table 3.

**Table 3:** The results of the Meta-analysis (for EH).

	**Allelic**				**Homozyous**				**Heterozyous**				**Dominant**				**Recessive**			
	***N***	**Sample (E/C)**	**OR (95%CI)**	***I*^**2**^**	***N***	**Sample (E/C)**	**OR (95%CI)**	***I*^**2**^**	***N***	**Sample (E/C)**	**OR (95%CI)**	***I*^**2**^**	***N***	**Sample (E/C)**	**OR (95%CI)**	***I*^**2**^**	***N***	**Sample (E/C)**	**OR (95%CI)**	***I*^**2**^**
Overall	18	10729/14579	0.81 (0.74, 0.90)	69%	18	10729/14579	0.69 (0.56, 0.85)	51%	18	10729/14579	0.81 (0.73, 0.89)	60%	20	11051/15926	0.79 (0.71, 0.87)	62%	18	10729/14579	0.77 (0.68, 0.86)	45%
All in HWE	16	10077/12972	0.83 (0.76, 0.92)	69%	16	10077/12972	0.69 (0.56, 0.85)	54%	16	10077/12972	0.84 (0.76, 0.92)	54%	16	10077/12972	0.82 (0.73, 0.91)	64%	16	10077/12972	0.77 (0.68, 0.86)	49%
Ethnicity																				
Chinese	12	6757/7838	0.83 (0.72, 0.96)	75%	12	6757/7838	0.71 (0.51, 0.98)	55%	12	6757/7838	0.81 (0.69, 0.95)	68%	12	6757/7838	0.80 (0.68, 0.95)	73%	12	6757/7838	0.72 (0.61, 0.85)	48%
Japanese	6	3972/6741	0.81 (0.73, 0.91)	53%	6	3972/6741	0.70 (0.53, 0.92)	49%	6	3972/6741	0.81 (0.72, 0.92)	40%	8	4294/8088	0.79 (0.71, 0.88)	29%	6	3972/6741	0.81 (0.69, 0.95)	46%
Gender																				
Male	10	3170/4706	0.72 (0.66, 0.78)	0%	10	3170/4706	0.55 (0.44, 0.68)	19%	10	3170/4706	0.68 (0.62, 0.76)	0%	11	3369/5732	0.67 (0.61, 0.73)	0%	10	3170/4706	0.65 (0.53, 0.80)	20%
Female	8	2538/3729	0.97 (0.84, 1.11)	52%	8	2538/3729	0.93 (0.77, 1.14)	5%	8	2538/3729	1.02 (0.92, 1.14)	48%	8	2538/3729	0.99 (0.82, 1.18)	52%	8	2538/3729	0.92 (0.75, 1.11)	0%
Alcohol consumption																				
Drinkers	4	2368/4340	0.71 (0.55, 0.92)	72%	4	2368/4340	0.60 (0.25, 1.47)	58%	4	2368/4340	0.70 (0.55, 0.89)	58%	5	2447/4514	0.72 (0.57, 0.91)	59%	4	2368/4340	0.71 (0.32, 1.55)	50%
No-drinkers	2	1026/918	1.19 (0.59, 2.40)	89%	2	1026/918	1.41 (0.34, 5.92)	85%	2	1026/918	0.98 (0.66, 1.44)	48%	3	1070/1065	0.95 (0.59, 1.52)	68%	2	1026/918	1.39 (0.39, 5.00)	82%
Source																				
Population	12	8761/10640	0.83 (0.77, 0.89)	46%	12	8761/10640	0.72 (0.62, 0.83)	14%	12	8761/10640	0.82 (0.75, 0.91)	47%	13	8960/11666	0.80 (0.73, 0.87)	45%	12	8761/10640	0.77 (0.68, 0.88)	9%
Hospital	6	1968/3939	0.86 (0.59, 1.25)	85%	6	1968/3939	0.85 (0.31, 2.32)	75%	6	1968/3939	0.79 (0.56, 1.13)	75%	7	2091/4260	0.80 (0.58, 1.11)	78%	6	1968/3939	0.92 (0.36, 2.33)	71%
Study design																				
Case-control	13	7018/8109	0.81 (0.71, 0.93)	74%	13	7018/8109	0.69 (0.51, 0.92)	51%	13	7018/8109	0.79 (0.68, 0.92)	68%	14	7217/9135	0.77 (0.67, 0.90)	71%	13	7018/8109	0.73 (0.56, 0.96)	43%
Cohort	5	3711/6470	0.84 (0.76, 0.93)	43%	5	3711/6470	0.72 (0.53, 0.97)	57%	5	3711/6470	0.85 (0.78, 0.94)	7%	6	3834/6791	0.83 (0.77, 0.91)	4%	5	3711/6470	0.76 (0.57, 1.02)	57%
Size																				
≥1000	8	8880/10477	0.85 (0.79, 0.90)	45%	8	8880/10477	0.71 (0.59, 0.84)	44%	8	8880/10477	0.85 (0.79, 0.92)	35%	9	9079/11503	0.83 (0.77, 0.89)	32%	8	8880/10477	0.75 (0.63, 0.89)	47%
<1000	10	1849/2112	0.76 (0.58, 1.01)	77%	10	1849/2112	0.68 (0.35, 1.33)	59%	10	1849/2112	0.72 (0.54, 0.95)	67%	11	1972/2433	0.72 (0.55, 0.95)	72%	10	1849/2112	0.76 (0.42, 1.39)	51%

**Figure 2:**
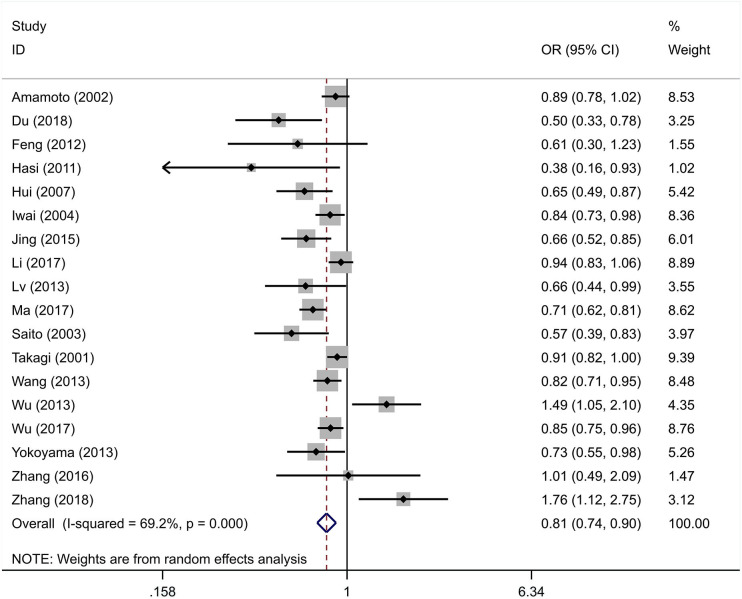
Forest graphs for the association between the ALDH-2 rs671 polymorphism and essential hypertension risk under the allelic model.

**Figure 3:**
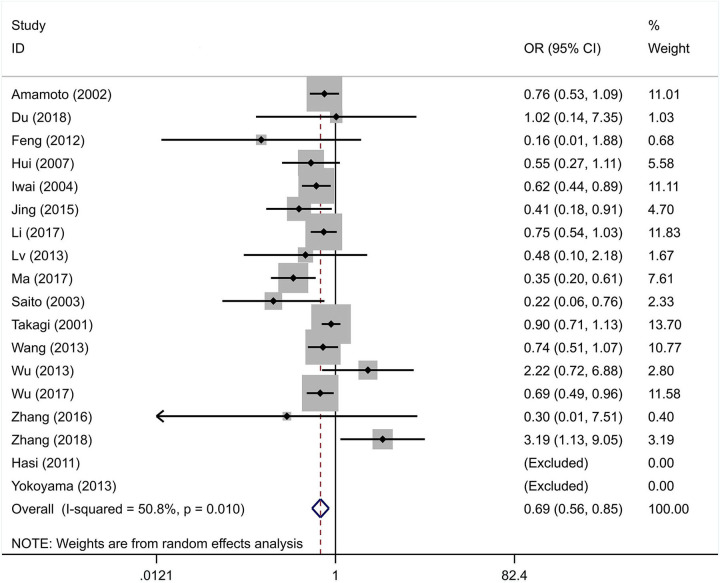
Forest graphs for the association between the ALDH-2 rs671 polymorphism and essential hypertension risk under the homozygous model.

**Figure 4:**
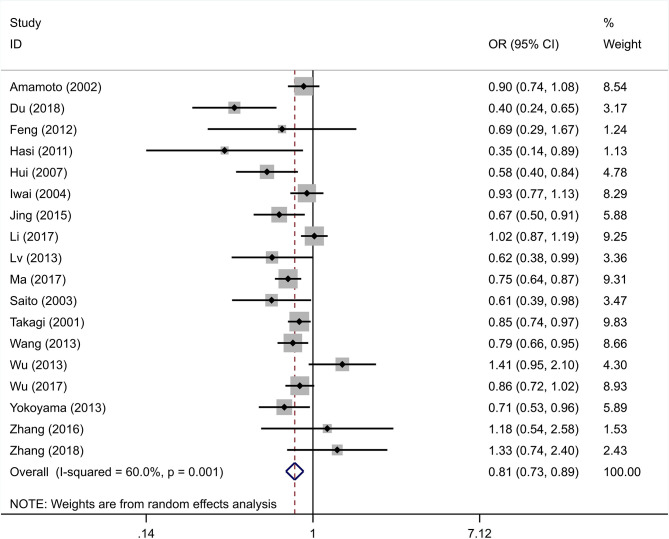
Forest graphs for the association between the ALDH-2 rs671 polymorphism with essential hypertension risk under the heterozygous model.

**Figure 5:**
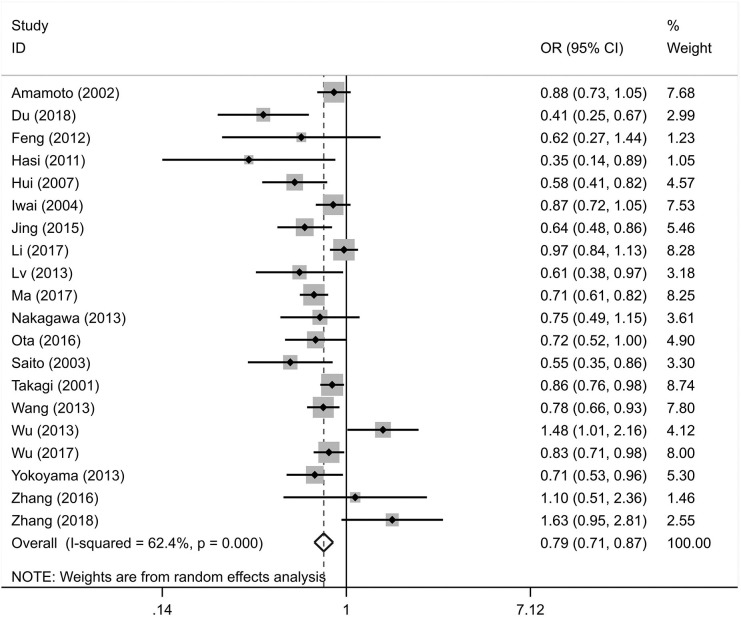
Forest graphs for the association between the ALDH-2 rs671 polymorphism and essential hypertension risk under the dominant model.

**Figure 6:**
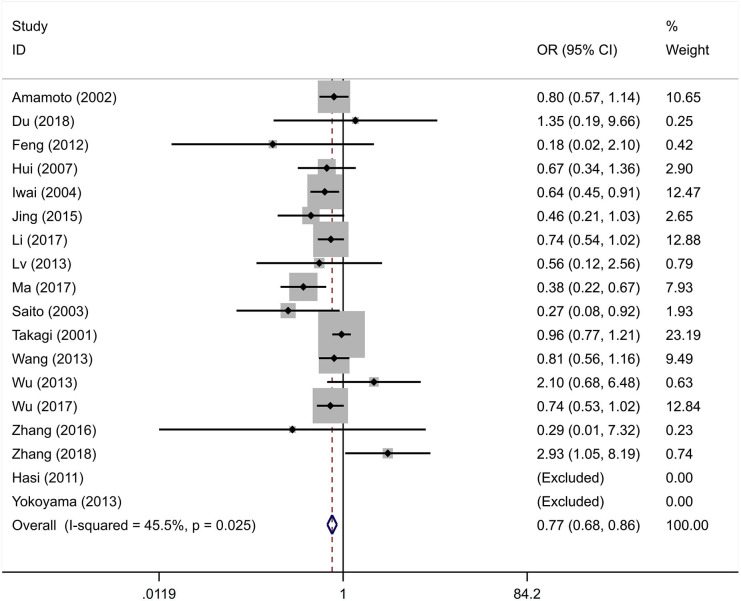
Forest graphs for the association between the ALDH-2 rs671 polymorphism and essential hypertension risk under the recessive model.

The integral analysis of 20 articles (Takagi et al., 2001; Amamoto et al., 2002; Saito et al., 2003; Iwai et al., 2004; Hui et al., 2007; Hasi et al., 2011; Feng et al., 2012; Lv et al., 2013; Nakagawa et al., 2013; Wang et al., 2013; Wu et al., 2013, 2017; Yokoyama et al., 2013; Jing et al., 2015; Ota et al., 2016; Zhang et al., 2016, 2018; Li et al., 2017; Ma et al., 2017; Du, 2018) revealed that a statistically significant association between the rs671 polymorphism and the risk of essential hypertension was observed under all models (Figures 2–6): allelic model (OR = 0.81, 95% CI: 0.74–0.90), homozygous model (OR = 0.69, 95% CI: 0.56–0.85), heterozygous model (OR = 0.81, 95% CI: 0.73–0.89), dominant model (OR = 0.79, 95% CI: 0.71–0.87), and recessive model (OR = 0.77, 95% CI: 0.68–0.86). In addition, an analysis of 16 articles (Takagi et al., 2001; Amamoto et al., 2002; Saito et al., 2003; Iwai et al., 2004; Hui et al., 2007; Hasi et al., 2011; Feng et al., 2012; Lv et al., 2013; Wang et al., 2013; Wu et al., 2013, 2017; Jing et al., 2015; Zhang et al., 2016, 2018; Li et al., 2017; Ma et al., 2017) in which the genotype distribution in the control group was in line with HWE revealed that the mutation of the rs671 polymorphism was associated with a significantly decreased risk of essential hypertension under all models: allelic model (OR = 0.83, 95% CI: 0.76–0.92), homozygous model (OR = 0.69, 95% CI: 0.56–0.85), heterozygous model (OR=0.84, 95% CI: 0.76–0.92), dominant model (OR = 0.82, 95% CI: 0.73–0.91), and recessive model (OR = 0.77, 95% CI: 0.68–0.86).

### Meta-Analysis for the Risk of Essential Hypertension: Stratified Analyses

Because the heterogeneity in the integral analyses was significant, a string of stratified analyses were implemented to determine the potential reasons for the between-study heterogeneity from other methodological aspects. In the stratified analyses, 20 articles were stratified by ethnicity, gender, alcohol consumption, source of control, study design, sample size, and genotyping methodology under the allelic, homozygous, heterozygous, dominant and recessive models (Table 3).

When all studies were stratified by ethnicity, there was a significant association between the rs671 polymorphism and EH risk in populations of Japanese descent under all models, and the same result was found in populations of Chinese descent. When stratified by gender, there was a significant association between the rs671 polymorphism and EH risk in men under all models; however, there was no significant association in women. When stratified by alcohol consumption, there was a significant association between the rs671 polymorphism and EH risk in drinkers under the allelic, heterozygous and dominant models; however, there was no significant association in non-drinkers. In the subgroup analysis stratified by the source of control, the EH risk prediction was significant in population-based studies for all models; however, the EH risk prediction was not significant in hospital-based studies for all models. When stratified by study design, the EH risk prediction was significant in case-control studies for all models; the EH risk prediction was only not significant in cohort studies for the recessive model. When stratified by sample size, the EH risk prediction was significant in studies with a sample size ≥ 1,000 for all models, and the EH risk prediction was significant in studies with a sample size <1,000 for the heterozygous and dominant models.

### Meta-Analysis for Blood Pressure Levels

Because of the low frequency of AA homozygotes and to avoid deviations from sample size, the association between the rs671 polymorphism and blood pressure levels was only investigated under the dominant model (A carriers vs. GG carriers). The detailed results of the WMDs and 95% CIs for different comparisons are shown in Table 4.

**Table 4:** The results of the Meta-analysis (for BP).

	**SBP**			**DBP**		
	***N***	**WMD (95%CI)**	***I*^**2**^**	***N***	**WMD (95%CI)**	***I*^**2**^**
Overall	12	−1.78 (−3.02,-0.53)	100%	12	−1.09 (−1.58, −0.61)	100%
Ethnicity						
Chinese	5	−0.57 (−1.17,0.04)	89%	5	−0.50 (−1.05,0.05)	97%
Japanese	7	−2.15 (−3.91, −0.39)	100%	7	−1.52 (−2.12, −0.93)	100%
Gender						
Male	4	−4.08 (−4.16, −3.99)	0%	4	−2.27 (−2.32, −2.22)	0%
Female	2	1.08 (1.01,1.15)	0%	2	−0.26 (−1.51,1.00)	76%
Alcohol consumption						
Drinkers	2	−2.71 (−7.51,2.09)	87%	2	−0.86 (−0.97, −0.75)	0%
Nodrinekers	2	−0.24 (−1.26,0.79)	45%	2	−0.13 (−0.20, −0.06)	19%

**Figure 7:**
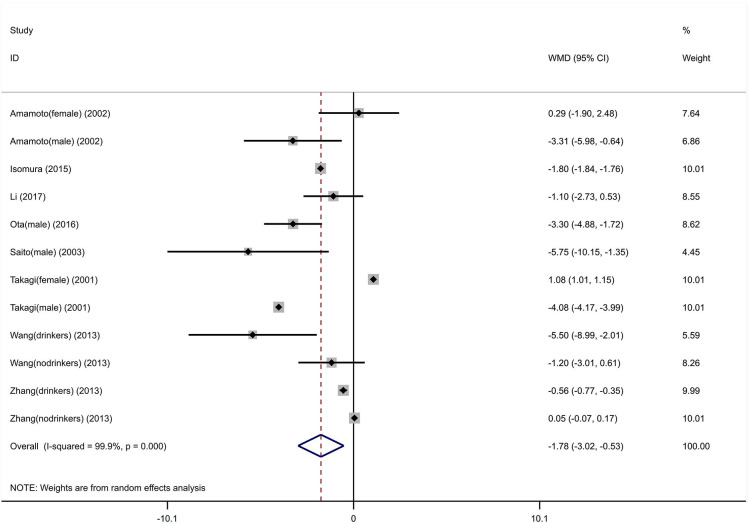
Forest graphs for the association between the ALDH-2 rs671 polymorphism and systolic blood pressure levels.

**Figure 8:**
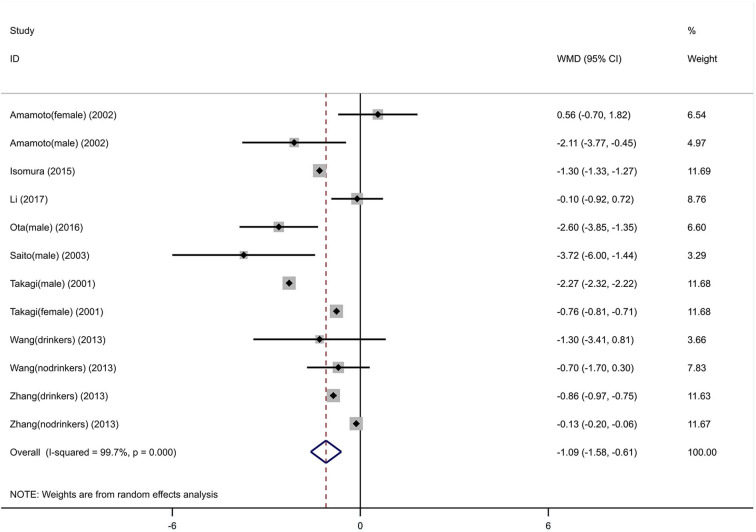
Forest graphs for the association between the ALDH-2 rs671 polymorphism and diastolic blood pressure levels.

An integral analysis of 12 individual studies (Takagi et al., 2001; Amamoto et al., 2002; Saito et al., 2003; Wang et al., 2013; Zhang et al., 2013; Isomura et al., 2015; Ota et al., 2016; Li et al., 2017) revealed significant variations in blood pressure between A carriers and GG homozygote carriers (Figures 7, 8). The A carriers had lower SBP (WMD = −1.78, 95% CI: −3.02 to −0.53) and DBP (WMD = −1.09, 95% CI: −1.58 to −0.61) levels than GG homozygote carriers. In the subgroup analysis stratified by gender, the significant variation in blood pressure between A carriers and GG homozygote carriers remained in men (SBP: WMD = −4.08, 95% CI: −4.16 to −3.99; DBP: WMD = −2.27, 95% CI: −2.32 to −2.22) but not in women (SBP: WMD = 1.08, 95% CI: 1.01–1.15; DBP: WMD = −0.26, 95% CI: −1.51–1.00). In the subgroup analysis stratified by alcohol consumption, the significant variation in DBP between A carriers and GG homozygote carriers remained in both drinkers (WMD=-0.86, 95% CI: −0.97 to −0.75) and non-drinkers (WMD = −0.13, 95% CI: −0.20 to −0.06); there was no significant variation in SBP in either drinkers (WMD=-2.71, 95% CI: −7.51–2.09) or non-drinkers (WMD=-0.24, 95% CI: -1.26–0.79).

### Sensitivity Analysis

The sensitivity analysis was performed by sequentially dropping one inter-study at a time to detect the influence of each inter-study on the summary OR and WMD. The outcomes of our meta-analysis were not altered greatly when each individual study was omitted, suggesting that the overall results were stable and robust.

### Publication Bias

**Figure 9:**
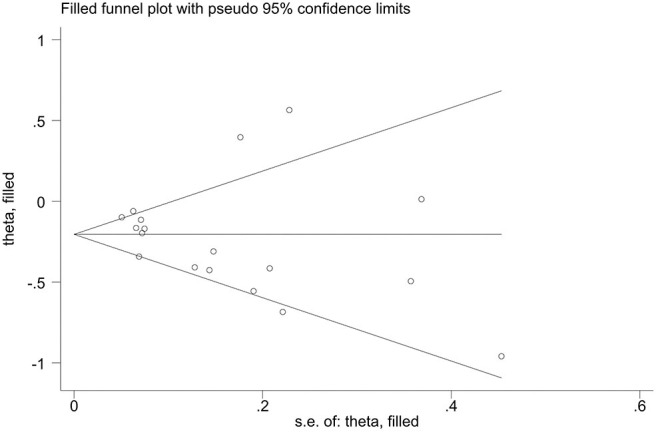
Filled funnel plots for the association between the ALDH-2 rs671 polymorphism and essential hypertension risk under the allelic model.

**Figure 10:**
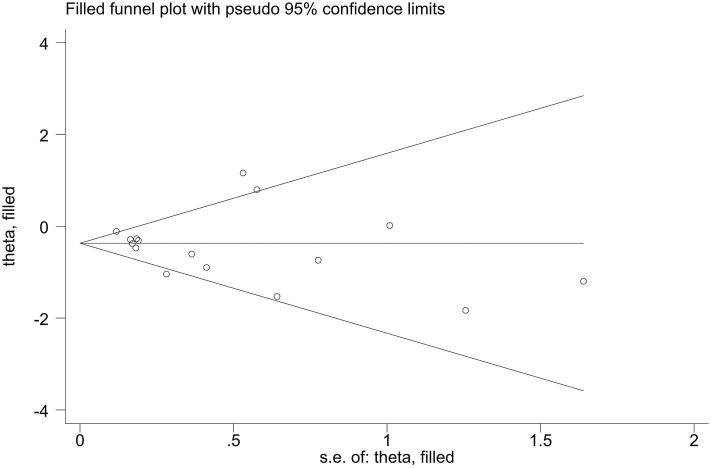
Filled funnel plots for the association between the ALDH-2 rs671 polymorphism and essential hypertension risk under the homozygous model.

**Figure 11:**
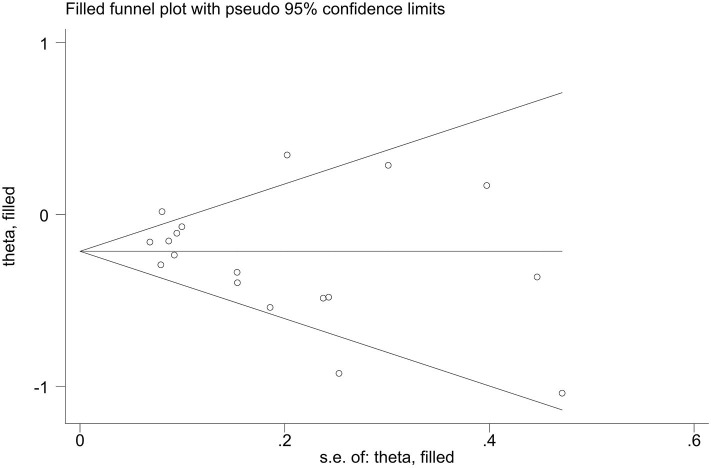
Filled funnel plots for the association between the ALDH-2 rs671 polymorphism and essential hypertension risk under the heterozygous model.

**Figure 12:**
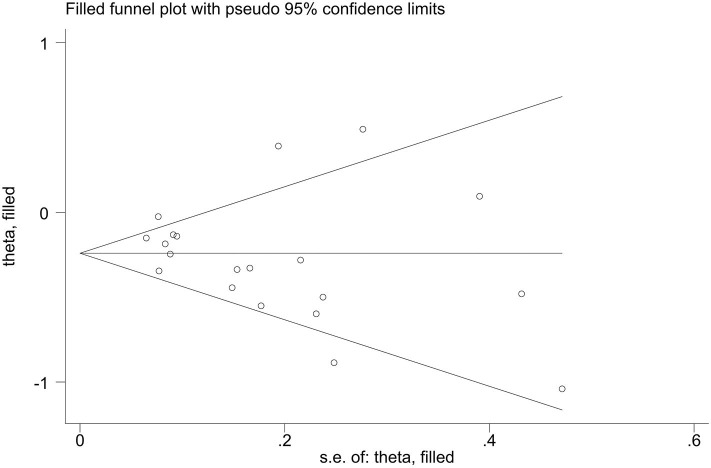
Filled funnel plots for the association between the ALDH-2 rs671 polymorphism and essential hypertension risk under the dominant model.

**Figure 13:**
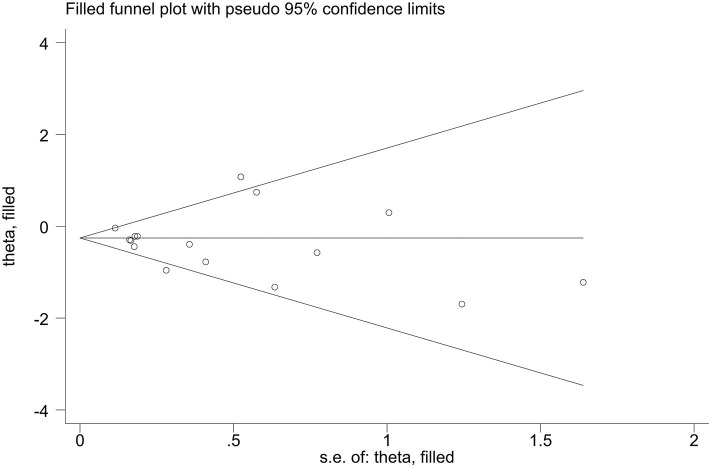
Filled funnel plots for the association between the ALDH-2 rs671 polymorphism and essential hypertension risk under the recessive model.

**Figure 14:**
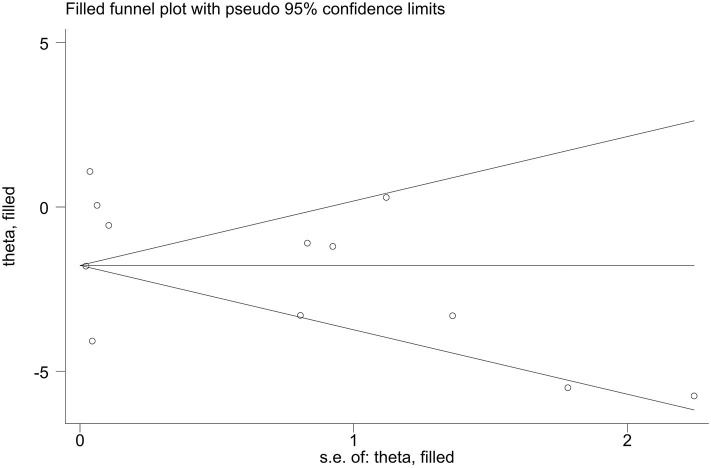
Filled funnel plots for the association between the ALDH-2 rs671 polymorphism and systolic blood pressure levels.

**Figure 15:**
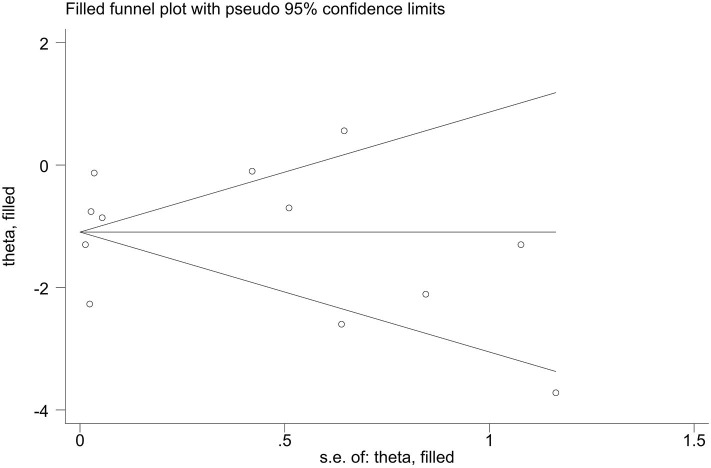
Filled funnel plots for the association between the ALDH-2 rs671 polymorphism and diastolic blood pressure levels.

Publication bias of the included studies was assessed using Begg's test, Egger's test and the trim-and-fill method. First, we applied Begg's test and Egger's test to evaluate publication bias. All *p*-values more than 0.05 was considered to have no evidence of publication bias (Table 5). The results of the trim-and-fill method were k = 0. The shape of the funnel plots of the trim-and-fill method in all comparisons did not show any obvious asymmetrical evidence (Figures 9–15), which revealed that there was little evidence of publication bias in the overall analysis.

**Table 5:** The results of Begg's test and Egger's test.

	**Allelic**	**Homozygous**	**Heterozygous**	**Dominant**	**Recessive**	**SBP**	**DBP**
Begg's test	0.363	0.444	0.363	0.206	0.392	0.537	0.304
Egger's test	0.305	0.390	0.216	0.224	0.429	0.902	0.698

## Discussion

Previous studies exploring the association between the ALDH-2 rs671 polymorphism and hypertension risk and blood pressure levels have provided controversial results, and the sample sizes in most of these studies were relatively small; thus, it was difficult to obtain credible genetic effects. Meta-analyses have been considered as one of the most important tools to precisely define the association between selected genetic polymorphisms and the risk for a morbid state. Based on this situation, we performed this study.

Our meta-analysis indicated that the ALDH-2 rs671 polymorphism is not only a major protective factor against the development of hypertension, particularly in males and drinkers, but it is also a critical factor in decreasing blood pressure. Based on the following aspects, we believe that our findings are more comprehensive and convincing. First, our meta-analysis incorporated more eligible studies, thus providing sufficient statistical power. Second, the association between the rs671 polymorphism and essential hypertension was investigated extensively with five genetic models. Third, we performed a series of more comprehensive subgroup analyses by factors addressed across different studies, which may influence the reliability. Fourth, the sensitivity analysis indicated that the results are stable and reliable. Finally, little evidence of publication bias was found in the overall analysis.

For essential hypertension association studies, a statistically significant association between the rs671 polymorphism and EH risk was observed under all models. Li et al. did a case-control study and meta-analysis, and shown that ALDH2 rs671 polymorphism may not associate with EH (Li et al., 2017). We have made a more comprehensive systematic analysis, and included the latest studies that they did not include. Therefore, we endorse our result even more. There was a common polymorphism of ALDH2 (rs671 G→ A) in East Asians. When stratified by ethnicity, significant associations were observed in both the Japanese and Chinese subgroups, suggesting that the association between the ALDH-2 rs671 polymorphism and the risk of essential hypertension did not differ between Chinese and Japanese populations. In the stratification analysis by gender, signification associations were found in males, although no significant associations were found in females. This may be due to physiological differences between males and females. Studies found that the female heart has elevated phosphorylation and ALDH2 activity (Lagranha et al., 2010). In the stratification analysis by alcohol consumption, signification associations were found in drinkers, although no significant associations were found in non-drinkers. This finding was not consistent with previous meta-analysis results (Fan et al., 2018). Zhang et al. concluded that the rs671 polymorphism may influence the risk of EH independent of alcohol consumption (Zhang et al., 2014). Compared with him, we added a 2018 study on the association between ALDH2 rs671 gene polymorphism and essential hypertension in non-drinkers (Zhang et al., 2018). We concluded that the rs671 polymorphism may affect the risk of essential hypertension in drinkers. Zhang et al. found that the hypertensive effect of alcohol was attributed to ethanol rather than acetaldehyde (Zhang et al., 2013). The rs671 polymorphism, G→ A, decreases the activity of alcohol-metabolizing enzymes. As a result, the rs671 polymorphism drinkers had less ethanol, which protected them from hypertension. In the subgroup analysis stratified by the source of control, the EH risk prediction was significant in population-based studies for all models, whereas the EH risk prediction was not significant in hospital-based studies for all models. We trusted that studies whose control groups were from populations accurately reflected the relationship between the rs671 polymorphism and EH risk. Finally, the study design and sample size did not alter the overall result.

For blood pressure association studies, a significant variation in blood pressure between A carriers and GG homozygote carriers was observed. This result was consistent with the results of the association between the rs671 polymorphism and EH risk. In the stratification analysis by gender, signification associations were found in the male subgroup for SBP and DBP levels, although no significant associations were found in females. In the stratification analysis by alcohol consumption, signification associations were found in both drinkers and non-drinkers' DBP levels, although no significant associations were found in SBP levels. Notably, there were relatively few studies based on gender differences and alcohol consumption differences; thus, it was difficult to obtain credible genetic effects.

However, there were several limitations in our study. First, only articles published in English and Chinese were incorporated, which led to a potential selection bias. Second, because of the lack of uniform background data for studies in meta-analyses, the data were not further stratified by other factors that may affect blood pressure such as salt consumption and smoking. Third, The ALDH2 polymorphism is observed to be associated with increased risk for diseases such as coronary artery disease and diabetics. However, due to the limitation of included articles, further stratification of the subjects according to these accompanied diseases could not be implemented in this study. Forth, As medications could affect the BP levels. However, due to the limitation of included articles, we were unable to analyze the use of drugs. Fifth, significant heterogeneity was detected even we performed subgroup analyses. Finally, the meta-analysis was limited by the inadequate sample size, particularly in the alcohol consumption subgroup analysis.

## Conclusions

The collective findings of this meta-analysis demonstrated that the mutation of the ALDH-2 rs671 polymorphism was significantly associated not only with a decreased predisposition toward essential hypertension but also with lowering blood pressure, suggesting that the ALDH-2 rs671 polymorphism might represent an important genetic marker of hypertension. These findings potentially further our understanding of the contributing role of the ALDH-2 rs671 polymorphism in blood pressure regulation and in the pathogenesis of hypertension.

## Data Availability Statement

All datasets generated for this study are included in the article/supplementary material.

## Author Contributions

YZhe, CN, and ZF conceived and designed the experiments. YZhe, XZ, and YL performed the experiments. YZhe, XZ, and LQ analyzed the data. YZhe and YZha contributed materials and analytical tools. YZhe, XZ, and CN wrote the manuscript. YZhe, JL, and ZF revised the manuscript. All authors reviewed and approved the manuscript prior to submission.

## Conflict of Interest

The authors declare that the research was conducted in the absence of any commercial or financial relationships that could be construed as a potential conflict of interest.

## References

[B1] Amamoto K. Okamura T. Tamaki S. Kita Y. Tsujita Y. Kadowaki T. (2002). Epidemiologic study of the association of low-Km mitochondrial acetaldehyde dehydrogenase genotypes with blood pressure level and the prevalence of hypertension in a general population. Hypertens. Res. 25, 857–864. 10.1291/hypres.25.857 12484509

[B2] Chen W.-W. Gao R.-L. Liu L.-S. Zhu M.-L. Wang W. Wang Y.-J. (2017). China cardiovascular diseases report 2015: a summary. J. Geriatr. Cardiol. 14, 1–10. 10.11909/j.issn.1671-5411.2017.01.012 28270835 PMC5329726

[B3] Chen W.-W. Gao R.-L. Liu L.-S. Zhu M.-L. Wang W. Wang Y.-J. (2018). China cardiovascular diseases report 2017: a summary. Chin. Circ. J. 33, 1–8.

[B4] DerSimonian R. Laird N. (1986). Meta-analysis in clinical trials. Control. Clin. Trials. 7, 177–188. 10.1016/0197-2456(86)90046-2 3802833

[B5] Du J.-Y. (2018). Correlation analysis of Aldehyde dehydrogenase 2 genetic Glu504Lys polymorphism and hypertension in chongqing area. Int. J. Lab. Med. 39, 950–952. 10.3969/j.issn.1673-4130.2018.08.015

[B6] Eriksson C. J. (2001). The role of acetaldehyde in the actions of alcohol. Alcohol. Clin. Exp. Res. 25, 15S–32S. 10.1111/j.1530-0277.2001.tb02369.x 11391045

[B7] Fan Y. Chen Z. Ye T. Lin W. Wang Q. Lin B. (2018). Aldehyde dehydrogenase II rs671 polymorphism in essential hypertension. Clin. Chim. Acta 487, 153–160. 10.1016/j.cca.2018.09.037 30273545

[B8] Feng J. Wang C. Ye Q. Yin Z.-Y. Guo A.-B. Huang M.-M. (2012). Relationship between gene polymorphism of acetaldehyde dehydrogenase 2 and hypertension in aged patients. Chin. J. Cardiovasc. Rehabil. Med. 21, 143–146. 10.3969/j.issn.1008-0074.2012.02.10

[B9] Hasi T. Hao L. Yang L. Su X. L. (2011). Acetaldehyde dehydrogenase 2 SNP rs671 and susceptibility to essential hypertension in Mongolians: a case control study. Genet. Mol. Res. 10, 537–543. 10.4238/vol10-1gmr1056 21476199

[B10] Higgins J. P. Thompson S. G. Deeks J. J. Altman D. G. (2003). Measuring inconsistency in meta-analyses. BMJ 327, 557–560. 10.1136/bmj.327.7414.557 12958120 PMC192859

[B11] Hui P. Nakayama T. Morita A. Sato N. Hishiki M. Saito K.. (2007). Common single nucleotide polymorphisms in Japanese patients with essential hypertension: aldehyde dehydrogenase 2 gene as a risk factor independent of alcohol consumption. Hypertens. Res. 30, 585–592. 10.1291/hypres.30.585 17785925

[B12] Hwang B.-F. Chang T.-Y. Cheng K.-Y. Liu C.-S. (2012). Gene-environment interaction between angiotensinogen and chronic exposure to occupational noise contribute to hypertension. Occup. Environ. Med. 69, 236–242. 10.1136/oemed-2011-100060 22107792

[B13] Ikeda N. Saito E. Kondo N. Inoue M. Ikeda S. Satoh T.. (2011). What has made the population of Japan healthy? Lancet 378, 1094–1105. 10.1016/S0140-6736(11)61055-6 21885105

[B14] Isomura M. Wang T. Yamasaki M. Hasan M. Z. Shiwaku K. Nabika T. (2015). Aldehyde dehydrogenase polymorphisms and blood pressure elevation in the Japanese: a cross-sectional and a longitudinal study over 20 Years in the Shimane CoHRE study. Dis. Markers. 2015:825435 10.1155/2015/825435 26185357 PMC4491569

[B15] Iwai N. Tago N. Yasui N. Kokubo Y. Inamoto N. Tomoike H.. (2004). Genetic analysis of 22 candidate genes for hypertension in the Japanese population. J. Hypertens. 22, 1119–1126. 10.1097/00004872-200406000-00012 15167446

[B16] Jia K. Wang H. Dong P. (2015). Aldehyde dehydrogenase 2 (ALDH2) Glu504Lys polymorphism is associated with hypertension risk in Asians: a meta-analysis. Int. J. Clin. Exp. Med. 8, 10767–10772. 26379870 PMC4565253

[B17] Jing C.-Q. Peng H. Li G.-Q. Dai X.-Y. (2015). Association between acetaldehyde dehydrogenase 2 gene rs671 polymorphism and essential hypertension in Han population from Xinjiang. J. Clin. Int. Med. 32, 174–177.

[B18] Kario K. (2015). Key points of the Japanese society of hypertension guidelines for the management of hypertension in 2014. Pulse 3, 35–47. 10.1159/000381300 26587456 PMC4646136

[B19] Lagranha C. J. Deschamps A. Aponte A. Steenbergen C. Murphy E. (2010). Sex differences in the phosphorylation of mitochondrial proteins result in reduced production of reactive oxygen species and cardio protection in females. Circ. Res. 106, 1681–1691. 10.1161/CIRCRESAHA.109.213645 20413785 PMC3127199

[B20] Li Z. M. Kong C. Y. Sun K. Y. Wang L. S. (2017). The ALDH2 gene rs671 polymorphism is not associated with essential hypertension. Clin. Exp. Hypertens. 39, 691–695. 10.1080/10641963.2017.1299749 28613083

[B21] Liberati A. Altman D. G. Tetzlaff J. Mulrow C. Gøtzsche P. C. Ioannidis J. P.. (2009). The PRISMA statement for reporting systematic reviews and meta-analyses of studies that evaluate healthcare interventions: explanation and elaboration. BMJ 339:b2700. 10.1136/bmj.b2700 19622552 PMC2714672

[B22] Lim S. S. Vos T. Flaxman A. D. Danaei G. Shibuya K. Adair-Rohani H.. (2012). A comparative risk assessment of burden of disease and injury attributable to 67 risk factors and risk factor clusters in 21 regions, 1990–2020: a systematic analysis for the Global Burden of Disease Study 2010. Lancet 380, 2240–2260. 10.1016/S0140-6736(12)61766-8 23245609 PMC4156511

[B23] Lv Y.-D. Hu X.-L. Wang Y.-Z. Xu W.-H. Tao P.-H. (2013). A study on the association between gene polymorphism of ALDH2(Glu504Lys) and hypertension among Chinese Han People in Zhejiang. Zhejiang Prev. Med. 25, 4–7. 10.3969/j.issn.1007-0931.2013.09.002

[B24] Ma C. Yu B.-X. Zhang W.-H. Wang W.-M. Zhang L.-P. Zeng L.-P. (2017). Associations between aldehyde dehydrogenase 2 (ALDH2) rs671 genetic polymorphisms, lifestyles and hypertension risk in Chinese Han people. Sci. Rep. 7:11136. 10.1038/s41598-017-11071-w 28894224 PMC5593832

[B25] Ma C. Yu B.-X. Zhang W.-H. Zeng Q. (2016). GW27-e0935 Associations between aldehyde dehydrogenase 2 (ALDH2) rs671 genetic polymorphisms, lifestyles and hypertension risk in Chinese Han people. J. Am. Coll. Cardiol. 68, C141–C142. 10.1016/j.jacc.2016.07.533 28894224 PMC5593832

[B26] Nakagawa T. Kajiwara A. Saruwatari J. Hamamoto A. Kaku W. Oniki K.. (2013). The combination of mitochondrial low enzyme activity aldehyde dehydrogenase 2 allele and superoxide dismutase 2 genotypes increases the risk of hypertension in relation to alcohol consumption. Pharmacogenet. Genomics 23, 34–37. 10.1097/FPC.0b013e32835b1707 23111423

[B27] Niu W.-Q. Qi Y. Hou S.-Q. Zhai X.-Y. Zhou W.-Y. Qiu C.-C. (2019). Haplotype-based association of the renin-angiotensin-aldosterone system genes polymorphisms with essential hypertension among Han Chinese: the Fangshan study. J. Hypertens. 27, 1384–1391. 10.1097/HJH.0b013e32832b7e0d 19412130

[B28] Ohsawa I. Kamino K. Nagasaka K. Ando F. Niino N. Shimokata H.. (2003). Genetic deficiency of a mitochondrial aldehyde dehydrogenase increases serum lipid peroxides in community-dwelling females. J. Hum. Genet. 48, 404–409. 10.1007/s10038-003-0046-y 12905081

[B29] Ota M. Hisada A. Lu X. Nakashita C. Masuda S. Katoh T. (2016). Associations between aldehyde dehydrogenase 2 (ALDH2) genetic polymorphisms, drinking status, and hypertension risk in Japanese adult male workers: a case–control study. Environ. Health Prev. Med. 21, 1–8. 10.1007/s12199-015-0490-2 26318866 PMC4693762

[B30] Perez-Miller S. Younus H. Vanam R. Chen C.-H. Mochly-Rosen D. Hurley T. D. (2010). Alda-1 is an agonist and chemical chaperone for the common human aldehyde dehydrogenase 2 variant. Nat. Struct. Mol. Biol. 17, 159–164. 10.1038/nsmb.1737 20062057 PMC2857674

[B31] Saito K. Yokoyama T. Yoshiike N. Date C. Yamamoto A. Muramatsu M.. (2003). Do the ethanol metabolizing enzymes modify the relationship between alcohol consumption and blood pressure? J. Hypertens. 21, 1097–1105. 10.1097/00004872-200306000-00009 12777946

[B32] Stang A. (2010). Critical evaluation of the Newcastle-Ottawa scale for the assessment of the quality of nonrandomized studies in meta-analyses. Eur. J. Epidemiol. 25, 603–605. 10.1007/s10654-010-9491-z 20652370

[B33] Takagi S. Baba S. Iwai N. Fukuda M. Katsuya T. Higaki J.. (2001). The aldehyde dehydrogenase 2 gene is a risk factor for hypertension in Japanese but does not alter the sensitivity to pressor e?ects of alcohol: the Suita study. Hypertens. Res. 24, 365–370. 10.1291/hypres.24.365 11510748

[B34] Wang Y. Zhang Y. Zhang J. Tang X. Qian Y. Gao P.. (2013). Association of a functional single-nucleotide polymorphism in the ALDH2 gene with essential hypertension depends on drinking behavior in a Chinese Han population. J. Hum. Hypertens. 27, 181–186. 10.1038/jhh.2012.15 22551939

[B35] Wu Y. Ni J. Cai X. Lian F. Ma H. Xu L.. (2017). Positive association between ALDH2 rs671 polymorphism and essential hypertension: a case-control study and meta-analysis. PLoS ONE 12:e0177023. 10.1371/journal.pone.0177023 28472173 PMC5417637

[B36] Wu Y.-Z. Li Y.-X. Zhao Q.-H. Guo Y.-J. Dang W. Qiao N. (2013). Association of acetaldehyde dehydrogenase 2 polymorphism with hypertension. Chin. Remed. Clin. 13, 1421–1422.

[B37] Xu T. Liu S.-Y. Ma T.-T. Jia Z.-Y. Zhang Z.-F. Wang A.-M. (2017). Aldehyde dehydrogenase 2 protects against oxidative stress associated with pulmonary arterial hypertension. Redox Biol. 11, 286–296. 10.1016/j.redox.2016.12.019 28030785 PMC5192477

[B38] Yokoyama A. Mizukami T. Matsui T. Yokoyama T. Kimura M. Matsushita S.. (2013). Genetic polymorphisms of alcohol dehydrogenase-1B and aldehyde dehydrogenase-2 and liver cirrhosis, chronic calcific pancreatitis, diabetes mellitus, and hypertension among Japanese alcoholic men. Alcohol. Clin. Exp. Res. 37, 1391–1401. 10.1111/acer.12108 23550892

[B39] Zhang L.-L. Dong L.-M. Ma Q. Yang G.-C. (2018). Association of ALDH2 rs671 polymorphism with essential hypertension: a case-control study in non-drinking Han Chinese. Int. J. Clin. Exp. Med. 11, 6222–6227.

[B40] Zhang S.-Y. Chan S.-W. Zhou X. Chen X.-L. Mok D. K. W. Lin Z.-X.. (2014). Meta-analysis of association between ALDH2 rs671 polymorphism and essential hypertension in Asian populations. Herz 40, 203–208. 10.1007/s00059-014-4166-2 25403981

[B41] Zhang W. S. Xu L. Schooling C.-M. Jiang C.-Q. Cheng K.-K. Liu B.. (2013). Effect of alcohol and aldehyde dehydrogenase gene polymorphisms on alcohol-associated hypertension: the Guangzhou Biobank cohort study. Hypertens. Res. 36, 741–746. 10.1038/hr.2013.23 23615284 PMC3734527

[B42] Zhang Z.-Y. Yu Y.-W. Li L. Liu T. Tang L.-N. (2016). The correlation between CASZ1, ZNF652, MTHFR, ATP2B1 and ALDH2 gene single nucleotide polymorphism and essential hypertension in Miao ethnic minority of Guizhou. Chin. J. Dis. Control. Prev. 20, 634–636. 10.16462/j.cnki.zhjbkz.2016.06.023

